# Blood Cancer Clinical Trials Long-term Follow-up Using Integrated Healthcare Systems Data (BLISS): protocol for a data-linkage study integrating randomised clinical trials with national healthcare systems data

**DOI:** 10.1136/bmjopen-2026-120416

**Published:** 2026-05-11

**Authors:** Lesley Smith, Janice Hoang, Sharon Gillson, Jeanine Richards, Gordon Cook, Sarah Brown, Kara-Louise Royle, Sadie Roberts, Catherine Olivier, Chris Parish, Katie Spencer, Shelagh Thompson, David A Cairns

**Affiliations:** 1 Leeds Cancer Research UK Clinical Trials Unit, Leeds Institute of Clinical Trials Research, University of Leeds, Leeds, UK; 2 NIHR Leeds Biomedical Research Centre, Leeds, UK; 3 Academic Unit of Health Economics, Leeds Institute of Health Sciences, University of Leeds, Leeds, UK; 4 National Disease Registration Service, NHS England, Leeds, UK; 5 Patient Representative, Basingstoke, Hampshire, UK

**Keywords:** Myeloma, Clinical Trial, Cancer, Health informatics

## Abstract

**Abstract:**

**Introduction:**

Randomised clinical trials (RCTs) are gold standard in evidence-based medicine, but follow-up typically relies on clinic visits and trial-specific data collection. Much of this information overlaps with routinely collected healthcare systems data (HSD), such as electronic health records and national registries. Leveraging HSD for trial follow-up has the potential to reduce cost, time and resource burden. However, concerns remain about data quality and evidence is needed to show that HSD-based outcomes are reported to an equivalent standard to trial-specific data.

The Blood Cancer Clinical Trials Long-term Follow-up Using Integrated Healthcare Systems platform will link data collected from multiple myeloma clinical trials with HSD to create a research database supporting extended follow-up and further methodological and clinical research.

**Methods and analysis:**

This data-linkage study includes participants from multiple myeloma RCTs conducted by the University of Leeds between 2008 and 2021. NHS (National Health Service) England will link these participants to HSD, including deaths and cancer registrations, systemic anticancer therapy, radiotherapy and Hospital Episode Statistics.

We will compare trial-collected outcomes with those derived from HSD, including mortality, treatment, second cancer incidence and major adverse events. Long-term overall survival will be estimated using national mortality data. HSD-derived demographic and clinical variables will be used to assess population representativeness relative to the wider myeloma population. Time to next treatment will be derived and evaluated as a surrogate for progression-free survival. HSD-derived frailty measures will be examined for prognostic utility, and radiotherapy and hospital records will be analysed to characterise bone-related treatments and skeletal complications.

**Ethics and dissemination:**

Ethical approval has been obtained from the East of England–Cambridge Central Research Ethics Committee, with Section 251 support from the Health Research Authority on advice from the Confidentiality Advisory Group. Findings will be disseminated through publications, conference presentations and engagement with stakeholders and patient groups.

**Trial registration:**

ISRCTN60123120, ISRCTN49407852, ISRCTN90889843, ISRCTN24989786, ISRCTN08577602, ISRCTN17354232,ISRCTN59395590, ISRCTN24593488, ISRCTN58227268, ISRCTN15028850.

**STRENGTHS AND LIMITATIONS OF THIS STUDY**

- Linkage to national, routinely collected healthcare systems data allow comprehensive evaluation of long-term outcomes beyond the original trial follow-up.
- Systematic assessment of data quality through direct comparison of trial-collected outcomes with national datasets strengthens the methodological validity of the linkage approach.
- Integration of multiple datasets (mortality, cancer registrations, treatment datasets and hospital admissions) supports analysis of a broad set of clinically relevant endpoints.
- Limitations include the current restriction of data linkage to England (with expansion to other UK nations planned), missing or limited personal identifiers that may reduce linkage completeness.
- Absence of primary care data and national routinely collected blood test restricts evaluation of comorbidities and community-based outcomes.

## Introduction

 Randomised controlled trials (RCTs) are the gold standard for evaluating treatment efficacy in evidence-based medicine. However, most trial follow-up relies on clinic visits and trial-specific data collection via case report forms (CRFs). These processes are resource intensive and typically limited to the funded research period, restricting the ability to assess long-term outcomes such as overall survival (OS) and late treatment effects. Prolonged follow-up is essential for understanding long-term benefit and harm, but traditional methods are costly and prone to loss to follow-up.

Healthcare systems data (HSD) including mortality records, cancer registrations, treatment data and hospital admissions offer a scalable and efficient means of extending follow-up. HSD can complement trial data, enable long-term follow-up and reduce cost and resource burden.^1^ Health Data Research UK has prioritised leveraging NHS-curated data to deliver faster, more efficient trials,^2^ a recommendation reinforced by the O’Shaughnessy Review of UK commercial clinical trials.^3^ However, the use of HSD for trial follow-up raises key concerns regarding data quality, completeness and reliability. Rigorous validation against trial-collected outcomes is essential before HSD can be adopted for regulatory or scientific use.^4 5^ While mortality and hospital episode datasets are well established for regulatory use,^6 7^ less is known about cancer registration and treatment data, particularly in haematological malignancies. Existing studies have evaluated HSD utility in breast,^8^ colorectal,^9^ ovarian^10^ and oesophageal cancers,^11^ there are currently no data evaluating its application for outcome ascertainment in clinical trials involving blood cancers.

To address this gap, we will develop the Blood Cancer Clinical Trials Long-term Follow-up Using Integrated Healthcare Systems (BLISS) platform to support the use of routinely collected HSD in multiple myeloma trials, enabling efficient post-trial follow-up, evaluation of data quality and additional clinical and scientific research. The platform will link participant data from mature clinical trials conducted at the University of Leeds Clinical Trials Research Unit with NHS England datasets, including mortality, cancer registrations, treatments and hospital activity.

Initial work will focus on trials with completed follow-up to assess patient-level and trial-level agreement and determine where HSD is sufficiently reliable to support future cost-efficient trial designs. Further research will target priority areas in contemporary myeloma trials, including frailty and bone disease. Once established, the BLISS platform can be expanded to include additional haematological malignancies and other routinely collected datasets.

### Objectives

This protocol outlines the establishment of the BLISS research database, with the initial research aims:

- To use routinely collected mortality data to assess long-term OS in a series of myeloma clinical trials.
- To conduct a series of data utility comparison studies (DUCkS) to assess the quality and reliability of HSD in comparison to trial-specific collected data relating to mortality, treatment during and after trial, second cancer incidence and adverse events including infection, fracture, venous thromboembolism and cardiovascular disease.
- To assess diversity and representativeness in trial participants and outcomes.
- To derive time to next treatment (TTNT) from HSD and evaluate if it can be used as a surrogate endpoint for progression-free survival (PFS) in myeloma clinical trials.
- To assess and compare methods of defining frailty based on HSD and assess the clinical outcomes and prognostic ability of these indices.
- To identify and describe bone-related treatment and outcomes as an exemplar of a key clinical outcome which has been difficult to assess in RCTs and where HSD leveraged through BLISS may provide insights and guide future trial development.

## Methods

### Study design

This is a data linkage study based on clinical trial data linked to national HSD. The BLISS project will run from May 2025 to April 2030. The BLISS platform will include trials that have completed their original planned follow-up and where no further data collections (via trial specific CRFs) are planned. Personal identifiers (NHS number, sex and date of birth) from trial participants will be sent to NHS England for linkage to national data sets on mortality, cancer registrations and treatment and Hospital Episode Statistics (HES). This protocol currently covers linkage to HSD in England, further linkage to data held in Wales and Scotland will be explored later and is subject to a substantial amendment to current ethical approvals.

### Study population and sample size

The study population will include participants enrolled into a series of multiple myeloma clinical trials conducted by University of Leeds CTRU (Clinical Trials Research Unit) between 2008 and 2021 that are now completed (N=5517 participants). The trials include both early-phase and late-phase trials in newly diagnosed and relapsed and refractory multiple myeloma. In total, 10 clinical trials evaluating widely adopted treatments in the UK will be incorporated. The corresponding treatments evaluated are summarised in table 1. Clinical trial data to be extracted for inclusion in the BLISS database will encompass baseline demographics, treatment details, response assessments, progression and survival outcomes and adverse events. These data will provide the reference standard against which outcomes derived from routinely collected HSD will be evaluated.

**Table 1:** Summary of included trials of treatment for multiple myeloma

Trial	Study period	N	Treatment combinations	Primary outcome	Available in NHS
Late-phase studies					
X* (REL, TE) ISRCTN60123120	2008–2012	297	PAD+HDM/(s) ASCT PAD+Intensive C-wkly	PAD effective in first relapse ASCT superior	Yes
XI* (TE) ISRCTN49407852	2010–2014	1512	CTD CRD CVD for poor responders HDM/ASCT R and RZ maintenance	CRD improves PFS and OS Addition of CVD improves PFS and OS R maintenance improves PFS and OS	Yes, except RZ
XI* (TNE) ISRCTN49407852	2010–2015	1852	CTDa CRDa CVD for poor responders R and RZ maintenance	R maintenance improves PFS	Yes, except RZ
XI+* (TE) ISRCTN49407852	2013–2016	1056	CTD CRD KCRD HDM/ASCT R maintenance	KCRD improves PFS	Yes, except KCRD
Early phase studies (REL)					
MUK1† ISRCTN90889843	2011–2012	95	Bendamustine, TD	Dose identified	No
MUK3† ISRCTN24989786	2012–2015	27	Panobinostat, tosedostat	Dose identified	Yes
MUK4† ISRCTN08577602	2013–2014	16	Vorinostat, VD	Improves response	No
MUK5† ISRCTN17354232	2013–2014	300	KCD	KCD non-inferior to CVD K maintenance improves PFS	Yes
MUK6† ISRCTN59395590	2013–2014	54	Panobinostat, VTD	Dose identified	Yes
MUK7† ISRCTN24593488	2016–2018	124	Pomalidomide, CD	Improves response	Yes
MUK8† ISRCTN58227268	2016–2018	113	Ixazomib, CD	Did not improve PFS	Yes
MUK12† ISRCTN15028850	2018–2021	71	Selinexor, CD	Did not improve PFS	Yes‡

* National Cancer Research Institute trial.

† Myeloma UK Clinical Trial Acceleration Network Early Phase Trial.

‡ Access through Cancer Drugs Fund.

CD, cyclophosphamide and dexamethasone; CRD, cyclophosphamide, lenalidomide and dexamethasone; CRDa, attenuated oral CRD; CTD, cyclophosphamide, thalidomide, and dexamethasone; CTDa, attenuated oral CTD; CVD, cyclophosphamide, bortezomib and dexamethasone; HDM/(s)ASCT, high-dose melphalan and (salvage) autologous stem cell support; KCD, carfilzomib, cyclophosphamide and dexamethasone; KCRD, carfilzomib, cyclophosphamide, lenalidomide and dexamethasone; PAD, bortezomib (PS-341) adriamycin and dexamethasone; PFS, progression-free survival; R, lenalidomide maintenance; REL, relapse (previously transplanted patients); RZ, combination lenalidomide and vorinostat maintenance; TD, bortezomib and thalidomide; TE, transplant eligible; TNE, transplant non eligible; VD, bortezomib and dexamethasone; VTD, velcade, thalidomide and dexamethasone.

Participants in these trials were recruited from NHS hospitals in England, Scotland and Wales. Initially the BLISS platform will focus on HSD from England, therefore participants will be included in the BLISS platform if their first treating hospital was in England, or if they transferred to an NHS hospital in England later in their treatment pathway.

### Healthcare systems data

All English HSD will be obtained from NHS England. These datasets will be analysed to compare a range of clinical trial outcomes and provide additional data that were not collected as part of the trial to allow for further clinical and methodological research as set out in the study objectives (table 2). The following datasets will be incorporated:

### Civil registration of death

**Table 2:** Summary of HSD datasets included in BLISS data linkage

Dataset name	Periods	Key variables	Purpose in study
Civil Registration of Deaths	2008 to latest available	Date of death, place of death, cause of death	Comparison with trial collected mortality and for long-term survival estimation
National Cancer Registration and Analysis Service	2008 to latest available	Cancer diagnosis, tumour characteristics, treatment details	Confirmation of cancer diagnosis and identify secondary cancers in our study population. Additional data items not part of trial-specific data collection (eg, ethnicity, deprivation and comorbidities)
Systemic Anti-Cancer Therapy (SACT)	2012 to latest available	Chemotherapy and systemic therapy records	Assess treatment exposure and patterns post-trial To be used to derive lines of therapy and time to next treatment. To be used in planned analysis of bone disease outcomes.
NDRS National Radiotherapy Dataset (RTDS)	2009 to latest available	Radiotherapy records	Assess treatment exposure and patterns post-trial. To be used in planned analysis of bone disease outcomes.
Hospital Episode Statistics (HES) Admitted Patient Care (APC), Outpatient Care (OP) Accident and Emergency (A&E) Emergency Care Data Set (ECDS)	APC and OP 2008/09 to 2024/25 A&E 2008–2009–2018/19, ECDS 2019/2020 to 2024/2025	Admissions, procedures, comorbidities	Capture comorbidities and complications relevant to survival. To be used to define frailty indices. Comparison with trial collected adverse events. Additional data items not part of trial-specific data collection (e.g. ethnicity, deprivation and comorbidities)

BLISS, Blood Cancer Clinical Trials Long-term Follow-up Using Integrated Healthcare Systems; HSD, healthcare systems data.

This dataset includes all formally registered and medically certified deaths in England and Wales, provided by the Office for National Statistics (ONS).^12^ It is recognised as complete and reliable and is widely used in trials where mortality is a primary endpoint. It will be used to obtain date, cause and place of death.

### Cancer registrations

Cancer Registrations are obtained from the National Disease Registration Service and provide comprehensive, quality-assured data on all cancer diagnoses in England.^13^ This dataset will be used to obtain diagnostic information, secondary tumours and variables that are not available in the trial datasets, including comorbidities, ethnicity and deprivation.

### Systemic anticancer therapy dataset

The systemic anticancer therapy (SACT) dataset includes information on chemotherapy prescriptions from April 2012,^14^ with mandatory data submission achieved across NHS trusts by July 2014. There are known gaps in haematological cancers, likely due to coding and complexity of treatment. As SACT has not been used and validated in blood cancer clinical trials, a comprehensive data extract will be included to enable mapping trial data to enable estimation of line of treatment (LOT) and TTNT.

### Radiotherapy dataset

The radiotherapy dataset (RTDS) is a mandatory dataset capturing all NHS-funded radiotherapy delivered in England from 2009 onwards.^15^ It includes information on the treatment intent, dose, fractionation and anatomical site treated. These data will support our planned research on myeloma bone disease.

### Hospital episode statistics

HES contains curated data informing NHS hospital activity in England, submitted for payment and monitoring. HES Admitted Patient Care provides inpatient hospital admissions data dating back to 1997/1998, including diagnosis, procedures, patient demographics, admission and discharge dates and hospital information.^16^ The Outpatient and A&E HES and Emergency Care Datasets will also be included to explore their additional value in evaluation of clinical trial endpoints. Diagnoses are coded using International Statistical Classification of Diseases and Related Health Problems revision 10 (ICD10)^17^ and operations and procedures are coded using Office of Population Censuses and Surveys Classification of Interventions and Procedures V.4 (OPCS-4).^18^ These will be used to understand hospital activity patterns during follow-up and adverse events during treatment.

### Data linkage

**Figure 1:**
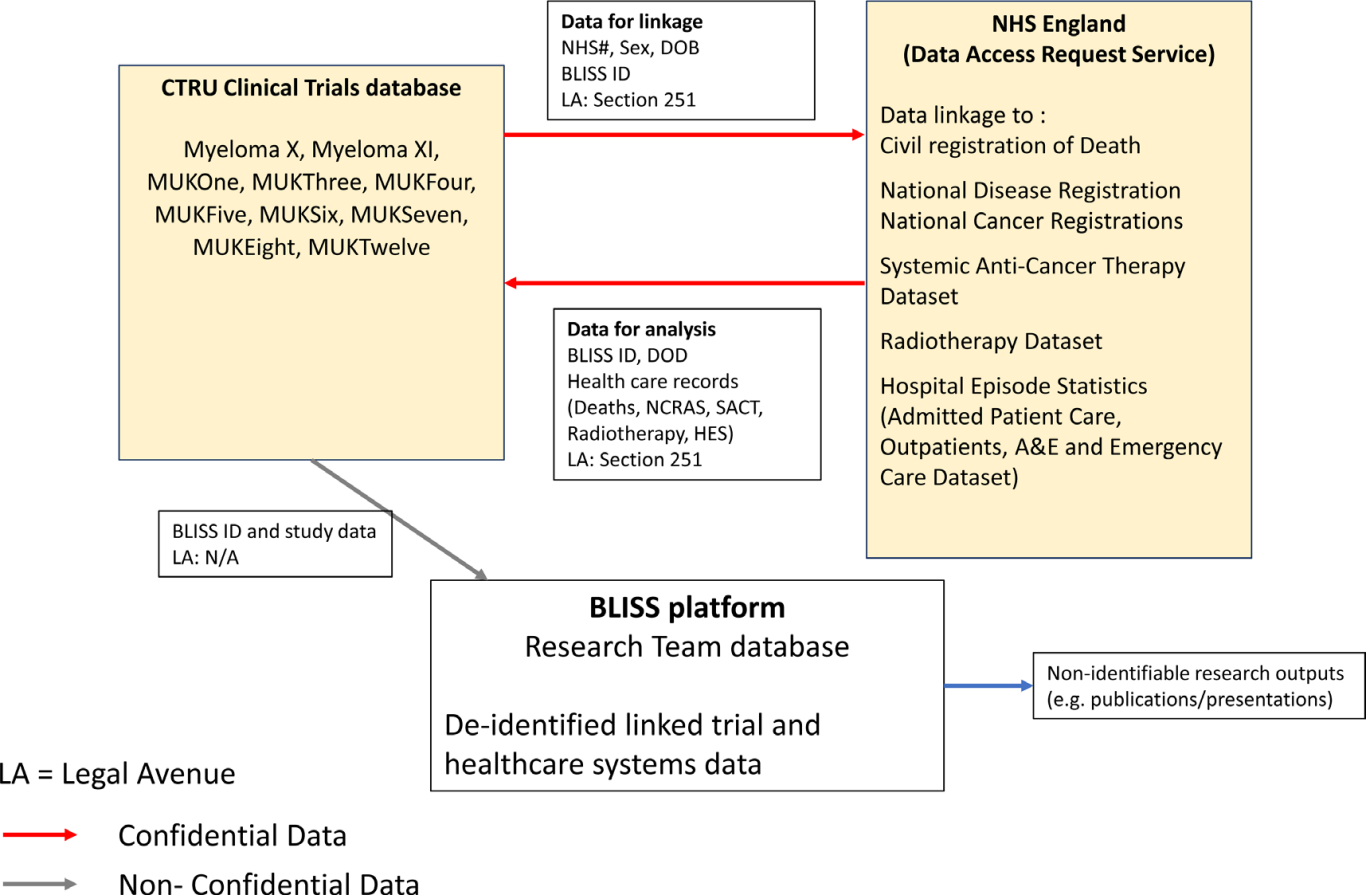
BLISS data linkage process. BLISS, Blood Cancer Clinical Trials Long-term Follow-up Using Integrated Healthcare Systems; DOB, date of birth, DOD, date of death; CTRU, Clinical Trials Research Unit; NCRAS, National Cancer Registration and Analysis Service; NHS, National Health Service; SACT, Systemic Anti-Cancer Therapy; HES, Hospital Episode Statistics.

Data linkage will be carried out by NHS England using exact matches on NHS number, sex and date of birth. Participants may be enrolled in more than one trial, therefore each individual will be assigned a unique BLISS ID. Leeds CTRU will securely transfer personal identifiers of the eligible trial cohort to NHS England. Following linkage, NHS England will return the linked datasets to the CTRU via encrypted files. Figure 1 shows the data flow diagram for the BLISS platform. The linked data will include all records from date of randomisation for each participant except for the Cancer Registration data, which will include the 6-month period prior to date of randomisation. HES data will be available up to 2024/2025, Mortality, Cancer Registration, SACT and RTDS will include the latest available data at the time of linkage, extending beyond the original trial follow-up.

### Statistical analysis plan

A deidentified, analysis-ready dataset integrating clinical trial data with linked HSD data will be created and managed in SAS (SAS Institute, Cary, North Carolina). Statistical analysis will be conducted in SAS and Stata (StataCorp V.2025. Stata Statistical Software: Release V.19. College Station, Texas: StataCorp LLC.).

### Linkage success and data quality checks

Data quality checks will be conducted once linked data are received from NHS England including reporting the number and proportion of trial participants successfully linked to each data source, overall and stratified by trial. Baseline demographics, characteristics and trial collected outcomes between those linked and non-linked participants will be described.

### Data utility comparison studies

A series of DUCkS will compare trial-collected outcomes with those derived from HSD to assess data quality and reliability. Outcomes include deaths, second cancers, trial treatment and adverse events such as infection, fracture, venous thromboembolism and cardiovascular disease. Adverse events will be identified using ICD-10 and OPCS-4 code lists. Mortality data will be sourced from national death registrations, second cancers from Cancer Registrations, trial treatment from SACT and adverse events from HES inpatient and outpatient datasets, with exploratory analyses using HES A&E and Emergency Care Data Set. Analyses will be conducted at both the patient level and the trial level. For each outcome (eg, death, second cancer, adverse event), patients will be categorised as outcome recorded in both data sources, outcome recorded in trial data only, outcome recorded in HSD only or outcome absent from both datasets.

Patient-level evaluation will use three predefined quality indicators: completeness, accuracy and agreement. Completeness will be quantified as the proportion of expected outcomes captured in each HSD source. Accuracy will be assessed using sensitivity and specificity. Sensitivity will be calculated as the true-positive rate, defined as the number patients with the outcome present in both HSD and the trial dataset divided by the total number of actual outcomes in the trial dataset. Specificity will be calculated as the number of patients with no outcome recorded in either dataset, divided by the number of patients with no outcome recorded in the trial dataset. Agreement between HSD and trial datasets will be measured using Cohen’s kappa. For outcomes present in both sources, event dates will be compared using median and interquartile differences, supported by graphical displays.

Trial-level analyses will be performed for outcomes recorded in both data sources. Event timing will be compared using restricted mean survival time to assess differences in survivor functions. Survival analyses will compare outcomes obtained from trial-collected follow-up with those produced under a scenario using only HSD-derived mortality. Treatment effects will follow each trial’s prespecified comparisons, with HRs estimated using appropriate Cox proportional hazards models. HRs from trial-based and HSD-based follow-up will be compared using absolute and relative differences with CIs. Kaplan-Meier (KM) curves will be generated for each treatment arm under both approaches, and forest plots will summarise HRs across trials to identify potential systematic differences. Subgroup analyses (eg, by trial or demographic characteristics) will be undertaken as appropriate.

### Long-term OS analyses

Long-term OS will be evaluated using HSD mortality data to extend follow-up beyond that available from trial-collected data. Multiple complementary analytical approaches will be applied to ensure comprehensive evaluation of survival outcomes including KM curves and Cox proportional hazards modelling. KM curves will be constructed for each individual trial, from which median OS and corresponding CIs will be derived. KM curves from trial-collected data alone and from trial plus HSD extended follow-up will be visually compared with evaluate differences in survival trajectory, late separation or convergence of curves and completeness of follow-up. Cox proportional hazards models will be fitted with appropriate covariate adjustment, and the proportional hazards assumption will be evaluated using Schoenfeld residuals and graphical diagnostics.

### Cure modelling

We will use statistical cure models as an alternative measure of long-term survival and estimate the cure fraction and characterise survival among uncured patients. Evidence for statistical cure will be evaluated through (1) a non-zero estimated cure fraction with corresponding CIs, (2) attenuation of the excess hazard to near zero beyond a clinically plausible timepoint and (3) the presence of a long-term survival plateau. Flexible parametric cure models within an excess-hazard (relative survival) framework will be used to separate disease-specific from background mortality.^19^ Background mortality will be derived from the ONS smoothed life tables, stratified by age, sex and deprivation. Models will adjust for key prognostic and clinical factors, including age, sex, performance status, ISS stage or cytogenetic risk, transplant eligibility, line of therapy, baseline biomarkers (eg, MRD, minimal residual disease) and randomised treatment where available.

### Representativeness and diversity analyses

We will evaluate the demographic and clinical representativeness of trial participants relative to the wider myeloma population using variables derived from HSD, including age, sex, ethnicity and deprivation (eg, IMD [Index of Multiple Deprivation] quintiles). Distributions of these characteristics will be described and compared with those of contemporaneous national myeloma cohorts identified through Cancer Registrations and HES, using standardised differences to quantify imbalance. We will also assess whether key demographic and clinical characteristics modify outcomes such as OS, identifying potential differential effects across population subgroups.

### Time to next treatment

TTNT will be derived from SACT and evaluated as a surrogate endpoint for PFS. TTNT could be a valid surrogate endpoint for PFS because it may be easier and inexpensive to measure routinely, especially from HSD. LOT will be identified from SACT using rules based on the interval between regimen changes and LOT index agents and verified using data from the clinical trials. Identifying the beginning of LOT following trial intervention allows estimation of TTNT, defined as time from enrolment to next treatment or death from any cause. Surrogacy assessment will follow recommended criteria requiring evidence of association at both the trial level, interventions that improve TTNT also improve PFS and the individual level, TTNT is prognostic for PFS after adjusting for treatment.^20^

Individual patient-level correlation between TTNT and PFS will be assessed using trial datasets linked with HSD. Treatment–effect correlation will be estimated via a meta-analytic approach.^21^ To assess individual-level surrogacy, Spearman’s ρ between TTNT and PFS will be estimated using KM estimates. The probability of outcome (PFS and TTNT) will be estimated, correlated across studies and weighted by the number of patients in each corresponding study.^22^ A joint-frailty copula model will also be fitted to further confirm individual patient-level correlation.^23 24^ Subgroup analyses, including by LOT and other clinically relevant factors, will be conducted where appropriate.

### Frailty assessment

Hospital admission and comorbidity data from HSD will be used to derive and externally validate several frailty assessment tools. The simplified International Myeloma Working Group frailty scale (sIMWGFS), based on age, Charlson Comorbidity Index, ECOG (Eastern Cooperative Oncology Group) performance status, will be constructed to generate a total risk score and classify patients as frail or non-frail.^25^ The addition of comorbidity data to Myeloma XI, the largest trial of its kind undertaken so far, will allow a direct comparison between sIMWGFS and the UK Myeloma Risk Profile.^26^ Several other frailty measures have been developed based on HES data including the Frailty Syndrome model,^27^ the Hospital Frailty Risk Score^28^ and the Secondary Care Administrative Records Frailty Index.^29^ These indices use ICD10 codes recorded in inpatient diagnostic fields and categorise the indices into three or four risk groups. External validation will assess associations between frailty classifications and key outcomes, including OS, PFS and early mortality. Model discrimination will be assessed by estimating Harrell’s C-statistic, D statistic alongside KM curves and HRs across frailty risk groups. Due to lack of data on baseline survival in the model development studies, assessment of model calibration will be limited.^30^

### Bone disease and outcomes

Treatments for myeloma bone disease include bisphosphonates (eg, zoledronic acid), monoclonal antibody therapy (eg, denosumab), radiotherapy and surgery. We will identify bone disease treatment use from SACT and pattern of use of radiotherapy (site, dose, utilisation) and surgery (procedure and use of postoperative SACT or radiotherapy). To understand the effects of bone disease treatment, we will derive skeletal-related events from HSD by building on work in prostate cancer.^31^ We will identify SREs based on ICD10 and OPCS-4 codes extracted in HES and RTDS relating to pathological fractures, spinal cord compression and needs for radiation or surgery. We will estimate the cumulative incidence of SREs with death treated as a competing risk and compare with outcomes from recent randomised trials and real-world evaluations. We will link the schedules of treatment given up to disease progression in each LOT and will assess clinical outcomes.

### Missing data

For each research question, patterns and mechanisms of missing data will be examined. Appropriate methods will be applied based on these assessments, including complete-case analysis where justified and multiple imputation for variables plausibly missing at random. Sensitivity analyses will be conducted to evaluate the robustness of findings to alternative missing data assumptions.

### Patient and public involvement

Patient and public involvement (PPI) was integral to the development of this study including early discussions with experienced PPI representatives and a focus group with myeloma patients. Across these consultations, patients expressed strong support for the project and emphasised the importance of generating up-to-date long-term survival information for people with myeloma. They endorsed the use of routinely collected data to monitor outcomes and highlighted key considerations for data governance and analysis, including the role of comorbidities and frailty.

To ensure ongoing PPI involvement, a patient with myeloma is a coinvestigator on the study and part of the Project Management Group (ST). We have established a PPI Advisory Group (PPIAG), consisting of six members, which will meet two times a year to provide guidance on study conduct, interpretation of findings and development of public-facing materials. Two patient representatives sit on the Project Steering Committee, which meets once a year to provide independent oversight and review progress.

## Ethics and dissemination

Ethical approval for a research database was obtained from the East of England—Cambridge Central Research Ethics Committee (25/EE/0099). The data linkage will be undertaken with ‘Section 251’ support from the Health Research Authority on advice from the Confidential Advisory Group (25/CAG/0061).

We will disseminate project progress and findings through conference presentations and peer-reviewed publications. A project website, hosted by the University of Leeds, will provide both research-facing and patient and public-facing content (https://ctru.leeds.ac.uk/bliss/). This platform will help keep trial participants and their families and carers informed about how their data are being used to address important research questions beyond the original trial aims. The website will also host links to publications and lay summaries developed in collaboration with the PPIAG. This will allow us to communicate results to researchers, PPI contributors, clinical collaborators, stakeholders and the wider public. Throughout the project, we will deliver a webinar series outlining research plans and emerging findings. To promote transparency and reproducibility, we will share code lists and analysis scripts via public repositories such as GitHub and provide links within corresponding publications. These materials will be openly accessible and easily searchable to support reuse by data providers and the wider research community.
